# Synthesis and characterization of magnetic nanocomposite for in vitro evaluation of irinotecan using human cell lines

**DOI:** 10.3906/kim-2010-53

**Published:** 2021-06-30

**Authors:** Tuba TARHAN

**Affiliations:** 1 Vocational High School of Health Services, Mardin Artuklu University, Mardin Turkey

**Keywords:** Drug nanocarrier, magnetic nanocomposite, cytotoxicity, glioblastoma multiforme, irinotecan

## Abstract

In this study, magnetic O-carboxymethyl chitosan (MOCC) nanocomposite was synthesized and characterized as a drug delivery system for loading the anticancer drug irinotecan (CPT-11). To increase the drug loading capacity, MOCC was synthesized by linking the carboxyl group functionally to chitosan. Also, several critical factors such as concentration, the dose of MOCC, and contact time for optimum drug loading condition were investigated. The loading capacity of CPT-11 onto MOCC was calculated as 5.6 mg/g, and the loaded drug concentration was calculated as 0.04787 mM at pH value of 5. Besides, the cytotoxic properties of MOCC, CPT- 11 loaded MOCC (MOCC-CPT-11), and free CPT-11 were studied on glioblastoma multiforme cell lines, including U87 and U373. According to the results, the MOCC-CPT-11 showed at least as toxic effect as free CPT-11 even at very low concentrations, while the MOCC showed slight toxicity (cell viability of 96% to 78%) on U373 cell lines at all concentrations and for 24 h and 48 h incubation times. Moreover, the results showed that the MOCC indicated significant toxicity in increasing concentrations and incubation times, and the MOCC-CPT-11 is as toxic as free CPT-11 on U87 cells at all concentrations and incubation times.

## 1. Introduction

Cancer is called a malignant tumor caused by the irregular division and proliferation of cells in a tissue or organ. Malignant glioma is the most common primary malignant brain tumor with a fatal clinical course and difficult to treat. Its applied treatment methods usually include chemotherapy, radiotherapy, and surgery. Chemotherapy is the core component in the treatment of malignant glioma. One of the most important problems of cancer chemotherapy nowadays is using anticancer drugs, which cannot recognize cancer cells solely and shows toxic effects on healthy cells as well. Besides, the administration of high doses is given to the body to provide a therapeutic dose concentration is causing severe side effects and systemic toxicity [1]. To improve the clinical results of chemotherapy it’s necessary to develop novel agents and new approaches. Nanotechnology and magnetic nanoparticles are one of them. The rapid advances in nanotechnology have brought important developments in the field of health as well. The usage and advantages of magnetic nanoparticles are being explored in the new generation of treatment methods for delivery to the target site and effective treatment without damaging healthy cells [1–3]. 

Irinotecan (CPT-11) is a water-soluble semisynthetic analogue of camptothecin (CPT), which inhibits the action of topoisomerase I. CPT-11 works by binding to the topoisomerase I-DNA complex, which prevents religation of the DNA strand. In this way, it causes double-strand DNA breakage, thereby triggering apoptotic cell death. This demonstrates cytotoxic activity against central nervous system tumor xenografts. CPT-11 is an anticancer drug that can cross the blood-brain barrier and is widely used in preclinical investigations. Moreover, CPT-11 has a lactone ring that is hydrolyzed in physiological pH and alkaline medium and transforms into a carboxylate form. The drug becomes toxic when converted to its unstable carboxylate form [4].

Casadó et al. investigated the cytotoxic effect of liposomal CPT-11 (CPT-11lip) on Hs68 and HeLa human cell lines for 0, 24, and 48 h to determine the suitability of CPT-11lip nanocarrier. According to the results of the MTT assay, the cell viability of HeLa cells decreased from 100% to 3% while Hs68 cells decreased from 100% to 40% after treatment of the CPT-11lip for 48 h [5]. Gao et al. carried out a comparison of the cytotoxic effects of nano Se, irinotecan and the combination of nano Se and irinotecan on HCT-8 tumor cells and normal IEC6 cells. When the combination nano Se and irinotecan (only at 10 mM concentration) was exposed on HCT-8 cells the cell viability of the HCT-8 decreased to 46%. On the other hand, the cell viability of the HCT-8 was found to lower than 62% and 80% for free irinotecan and nano Se alone, respectively. Furthermore, the nano Se, irinotecan and the combination of nano Se and irinotecan showed increasing cytotoxic effects on HCT-8 cells compared to IEC6 cells [6].

Due to its biocompatibility and biodegradability, chitosan and its many derivatives have been used extensively in biomedicine. However, one of the disadvantages of chitosan is that it cannot dissolve in water and only dissolves in an acidic solution (pH < 6). This restraint limits applications of chitosan in biomedicine. O-carboxymethyl chitosan (OCC) is one of the hydrophilic derivatives of chitosan. It has carboxyl groups as well as hydroxyl groups, thus it is better than chitosan to disperse in neutral and alkaline solutions. This property of OCC promises potential applications in biomedical ﬁelds [7–11]. 

Among many drug delivery systems, magnetic iron oxide nanoparticles stand out with their high drug loading capacity as well as targeting abilities, which stem from their magnetic field. It is thought that these nanoparticles can provide high therapeutic efficacy thanks to their magnetic and biocompatible properties [12]. Besides this, bare magnetic nanoparticles can be coated with suitable polymers to greatly reduce their toxicity for use in drug delivery systems. Polymer coated nanocarriers can be targeted to the desiring direction by the magnetic field applied from outside. On the other hand, in contrast to conventional chemotherapy, only tumor cells can be interfered with this method, and the effect of the drug on healthy cells is reduced [13]. Moreover, Allen et al. have noted that the drug loading capacity of the nanoparticles is highly correlated with the drug loading affinity of the polymer matrix. They stated that the presence of PCL-COOH increases the interaction between the polymer matrix and the loaded drug, thus providing a lower release rate [14].

Fe_3_O_4_ nanoparticles (NPs) were coated with chitosan, OCC, and n-succinyl-o-carboxymethyl chitosan polysaccharides by Zhu et al. They loaded CPT, known as an anticancer drug, on these different surfaces and incubated them for 24 h. Afterward, they investigated the release properties of CPT on 7721 liver cancer cells in vitro. According to the cytotoxicity assay results, polysaccharides modified with Fe_3_O_4_ NPs have not shown any cytotoxic effect against 7721. However, CPT loaded polysaccharide modified with Fe_3_O_4 _NPs have indicated a significant effect against 7721 liver cancer cells in comparison with free CPT [8].

In this study, MOCC was prepared and characterized to carry the CPT-11 anticancer drug. The drug loading capacity (CPT-11) and drug loading efficiency (%) onto MOCC were calculated as 5.6 mg/g and 47.87%, respectively at pH 5. Initial drug concentration and the loaded drug concentration were calculated as 0.10 mM and 0.04787 mM at pH 5, respectively. Also, the cytotoxic properties of the MOCC, CPT-11 loaded MOCC, and free CPT-11 on glioblastoma multiforme cell lines were investigated. While the MOCC showed only slight toxicity in increasing concentrations on the U373 cell line for 24 h and 48 h incubation times, the MOCC-CPT-11 indicated as high toxicity as free CPT-11 at all concentrations and incubation times. Moreover, the MOCC showed increased toxicity due to increasing concentration and incubation times on the U87 cells. Besides, the MOCC-CPT-11 indicated as high toxicity as free CPT-11 at all concentrations and incubation times on the U87 cell line.

## 2. Materials and methods

### 2.1. Chemicals

FeCl_3_.6H_2_O (ACS reagent, 97%) and FeCl_2_.4H_2_O (purists. p.a., ≥ 99.0% RT) were obtained from Sigma-Aldrich (Munich, Germany). NH_3_ (anhydrous, ≥ 99.98%), bromoacetic acid (reagent grade, 97%), and chitosan (low molecular weight) were purchased from Sigma-Aldrich. Acetic acid (glacial, 100%) was obtained from Merck (Kenilworth, NJ, USA). Sodium hydroxide (NaOH) and hydrochloric acid (HCl) were used to adjust the pH. Phosphate buffered saline was obtained from Sigma-Aldrich (100 tablet). The cellular studies of the irinotecan loaded MOCC were tested on glioblastoma multiforme cell lines (GBM), comparatively. The glioma cell lines U373 (ATCC) and U87 (ATCC) were grown in RPMI 1640 and Dulbecco’s modified eagle’s medium (DMEM) (Gibco—Invitrogen Corp., Dublin, Ireland), respectively. All media were supplemented with 10% fetal bovine serum (FBS), and the cell cultures were maintained at 37 °C in a humidified atmosphere of 5% CO_2_ in 95% air. All other chemicals were of reagent grade and used without further puriﬁcation. Deionized water was used in all of the experiments.

### 2.2. Instruments and measurements

The synthesized MOCC nanocomposite was characterized by fourier transform infrared spectroscopy (FT-IR, Thermo Scientific Nicolet 95 IS10 spectrometer), vibrating sample magnetometer (VSM, Cryogenic Limited PPMS), and X-ray photoelectron spectroscopy (XPS, PHI 5000 Versa Probe). The morphology and particle size of synthesized MOCC nanocomposite were examined by using transmission electron microscopy (TEM, JEOL mark JEM 2100F), high-resolution scanning electron microscopy (SEM, QUANTA 400F Field Emission), and dynamic light scattering spectrometer (DLS, MALVERN mark Zetasizer Nano ZS). Residual concentrations of the drug (CPT-11) were determined by using a UV/visible spectrophotometer (Perkin Elmer Lambda 25, Waltham, MA, USA) at 380 nm. The cytotoxicity results were determined with an optical density microplate reader (KCjunior ELISA) at 450 nm. 

### 2.3. Synthesis steps of the MOCC

**Figure 1:**
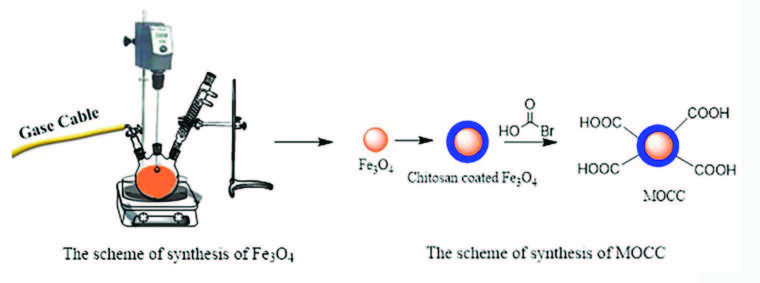
Representation of the scheme of reaction steps.

A series of reactions are necessary for the synthesis of MOCC. Firstly, iron oxide nanoparticles (Fe_3_O_4_ NPs) were synthesized by using iron salts mole ratio of 1:2 (0.86/2.36 g) FeCl_2_.4H_2_O/FeCl_3_.6H_2_O, which completely dissolved in 30 mL of deionized water with a mechanical stirrer under N_2_ in a 250 mL three-necked round bottom flask. This mixture was stirred in an oil bath under reflux up to 80 °C . Afterward, 28% 1.2 mL NH_3_ solution was added dropwise to the reaction medium and the resulting Fe_3_O_4_ NPs were precipitated and removed with an external magnet from the reaction medium [2]. Furthermore, 2 g of chitosan was dissolved in 1.5% acetic acid and interacted with the synthesized Fe_3_O_4_ NPs under a mechanical stirrer for 24 h at room temperature [7,15]. After 24 h, chitosan-coated Fe_3_O_4_ NPs was precipitated with a magnet and washed with deionized water several times. For the synthesis of MOCC, chitosan-coated Fe_3_O_4_ NPs were completely dispersed in enough deionized water. After then 6 g NaOH was dissolved in the ratio of 1:1 (V/V) isopropyl alcohol and deionized water and added to the dispersion and stirred with a mechanical stirrer in an oil bath under the reflux for 1 h at 50 °C. Afterwards, 8 g monobromo acetic acid was dissolved in 12 mL isopropyl alcohol and added to the reaction medium, and stirred for 4 h at 50 °C. After 4 h the precipitate, MOCC, was washed with 70% ethanol and then deionized water several times [7,16,17]. The reaction steps were schematized in Figure 1.

### 2.4. CPT-11 loading studies 

In CPT-11 loading studies, 0.5 mM CPT-11 stock solution was prepared in deionized water and stored in the dark at –18 °C . CPT-11 loading studies were carried out at a pH value of 5 on to synthesized MOCC. Due to the antitumor activity of CPT-11 increasing at a low pH, the drug loading studies were conducted at pH 5 [4]. The percent drug loading efficiency of MOCC was determined using the following equation (1).

(1)Drug loading efficiency %=Initial drug concentration (mM) - Supernatant drug concentration (mM)Initial drug concentration (mM)(x) 100

 (1)

**Figure 2:**
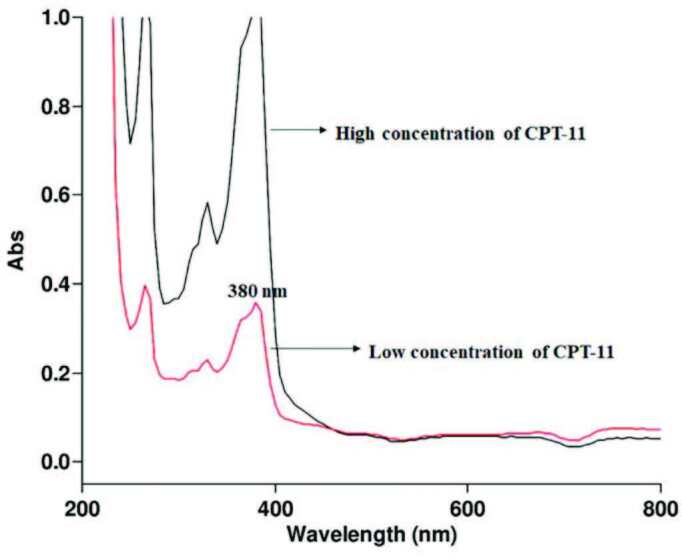
The UV spectrum of CPT-11 in PBS at pH 5.

In drug loading experiments, 0.50 mM stock drug solution was diluted to 0.10 mM with 2 mL phosphate buffer (pH = 5). Afterward, it interacted with 10 mg MOCC in the incubator at 100 rpm and 25 °C , and the drug-loaded MOCC nanocomposites were separated from the solution with an external magnetic field and washed with deionized water. These nanocomposites were stored dark at –18 °C for use in cytotoxicity experiments. In all experiments, the supernatant concentration was measured at the wavelength of 380 nm by using a UV spectrometer to determine the loaded drug concentration on the MOCC by comparing with a standard curve [18]. The UV spectrum of CPT-11 was shown in Figure 2 in PBS at pH 5. The drug loading efficiency (%) was calculated using equation (1). Also, in this study, the various critical parameters such as interaction time between CPT-11 and the MOCC nanocomposite, the effect of concentration of CPT-11, and the dose of MOCC on loading efficiency were investigated.

### 2.5. In vitro cytotoxicity studies 

The effect of MOCC, CPT-11 loaded MOCC, and free CPT-11 on cell viability was comparatively determined with a WST-8 colorimetric assay on cancerous U87 and U373 cell lines. First, glioblastoma multiforme cell lines were seeded at 8 × 10^3^ cells/well in a 96-well plate and incubated for 24 h. Afterward, the cell lines were treated with MOCC, free CPT-11, and CPT-11 loaded MOCC at the 5 different concentrations (0.2, 2, 10, 20, and 40 μM) in triplicates, and the cell lines were incubated for 24, 48, and 72 h. After each incubation time (24, 48, and 72 h), the cell lines were washed with PBS, then the fresh culture medium containing WST-8 reagent with a 1:10 ratio was added and incubated at 37 °C for 4 h. The percentage of living cells was evaluated by measuring the absorbance of formed formazan salts at 450 nm with an ELISA plate reader. 

### 2.6. Statistical analysis 

The results were represented as the mean ± standard deviation (SD) of three independent experiments. The data were analyzed by the student’s t-test and p < 0.05 was considered to be statistically significant.

## 3. Result and discussion 

### 3.1. Characterizations

**Figure 3:**
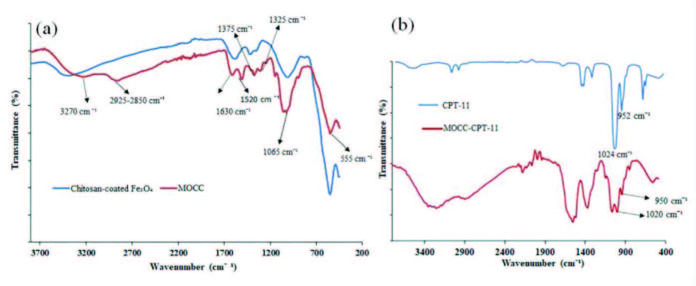
Comparatively FTIR spectra of chitosan coated Fe3O4 and MOCC (a), and CPT-11 and MOCC-CPT-11 (b).

The FT-IR spectra of MOCC and chitosan-coated Fe_3_O_4_ NPs were shown comparatively in Figure 3a. The wide and strong band at 3381 cm^−1^ showed the presence of –OH and –NH_2_ groups in the FT-IR spectrum of the chitosan-coated Fe_3_O_4_ NPs. In this spectrum, the stretching vibration peak of C–H and the vibration peak of -C-O were found at 2890 cm^−1 ^and1090 cm^−1^,respectively. Also, the characteristic amine deformation and the C-N stretching vibration peaks were observed corresponds to at 1634 cm^−1 ^and 1440 cm^−1^, respectively. Besides, the characteristic peak of Fe-O was demonstrated at 550 cm^−1^ [19–21]. Moreover, in the FT-IR spectrum of MOCC, the broadband at 3270 cm^−1 ^showed the O–H stretching vibration, and the stretching peak of -C-H was found between 2925 and 2850 cm^−1^. The stretching vibration peak of -COO at 1630 cm^−1^, thecharacteristic peak of –NH_3_^+^ absorption group at 1520 cm^−1^,and the absorption peak of –COO group between at 1375 and 1315 cm^−1^ were found to be. However, these peaks were not observed on the spectrum of chitosan-coated Fe_3_O_4_ NPs. This indicates that the MOCC was successfully synthesized from the chitosan. Besides, the peak of alcoholic hydroxyl -C–O stretching was represented at 1065 cm^−1^, and the characteristic peak of Fe-O was observed at 555 cm^−1^ in the MOCC spectrum [9,22–24]. Furthermore, as seen in Figure 3b, the C–C stretching vibration of CPT-11 was observed at 1024 and 950 cm^−1^ [25], and these peaks also were observed in the FTIR spectrum of MOCC-CPT-11. Moreover, an increase in the intensity of peaks between 1630 and 1375 cm^−1^ was observed. This indicated that CPT-11 was successfully loaded onto the MOCC.

**Figure 4:**
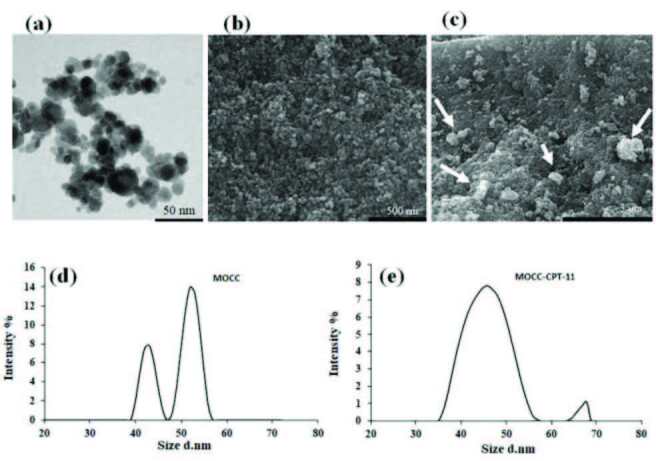
TEM image of MOCC (a). SEM images of MOCC (b) and MOCC-CPT-11 (c), and DLS measurement of MOCC (d) and MOCC-CPT-11 (e).

In Figure 4, TEM and SEM images gave information about the morphology and size of the MOCC. As can be seen TEM image, the MOCC particles showed a spherical structure, and also, the particle size of MOCC was measured as 13 ± 2.3 nm at 50 nm scale in Figure 4a. The particle size of the MOCC was found ideal for drug delivery systems. In Figure 4b, the SEM image of MOCC indicated that the nanoparticles were aggregated and had very small sizes. Furthermore, after the loading CPT-11, brightly colored CPT-11 particles were observed on some parts of the surface of MOCC in Figure 4c. Besides this, DLS measurements of nanoparticles were taken before and after loading the drug. Due to high agglomeration of the magnetic nanoparticles, it showed polydispersity as can be seen in Figures 4d and 4e. Accordingly, the particle sizes increased after the loading CPT-11 on MOCC in Figure 4e. Therefore, the particle size in the measurement of DLS was found to be much higher than the measurement of TEM.

**Figure 5:**
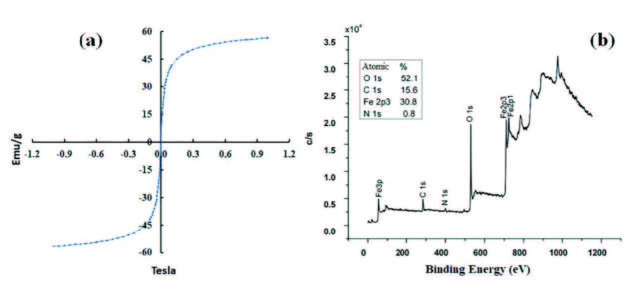
The magnetization value of the MOCC-CPT-11 (a), and the general XPS spectrum of MOCC (b).

The magnetization value of the CPT-11 loaded MOCC was measured by the change of the magnetic field versus magnetization at ambient conditions. In Figure 5a, the magnetization value of the CPT-11 loaded MOCC was determined as 56.0 emu/g. This value of magnetization was found to be ideal for drug delivery systems.

**Figure 6:**
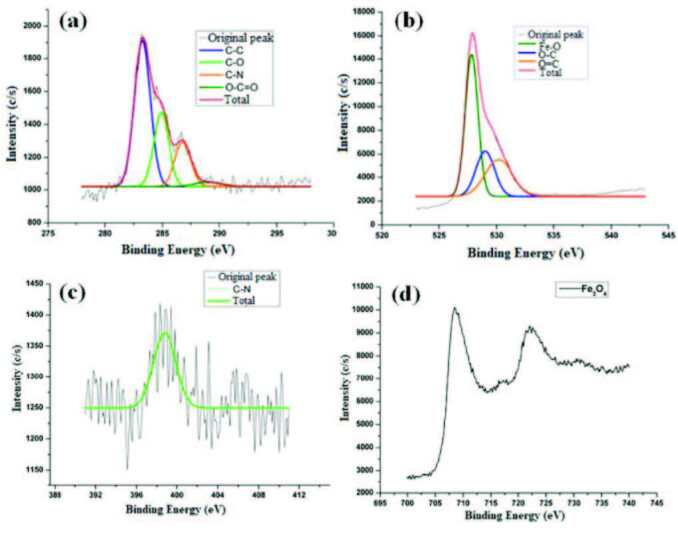
The high-resolution C(1s) (a), O (1s) (b), N (1s) (c), and Fe 2p (d) XPS spectra of MOCC.

Moreover, XPS spectra were also used to illuminate the MOCC structure. As seen in Figure 5b, in the general spectrum of MOCC, the atomic percentage of O (1s), C (1s), N (1s), and Fe (2p3) was measured as 52.1%, 15.6%, 0.8%, and 30.8%, respectively. Figure 6a showed the high-resolution spectrum of C (1s). In this spectrum, the peaks at ~284.4, ~286.4, ~287.1, and ~288.5 eV were attributed to the binding energy of C-C, C-O, C-N, and O-C=O, respectively [26,27]. Moreover, the high-resolution spectrum of O (1s) was shown in Figure 6b. Accordingly, the peaks at ~528.0, ~530.5, and ~531.5 eV corresponded to the binding energy of the O-Fe, O-C, and O=C, respectively [28,29]. Furthermore, in the high-resolution spectrum of N (1s), the peak of the C-N bond was observed at ~ 398.1 eV binding energy in Figure 6c [27]. And also, the spectrum of Fe 2p showed that the peaks of Fe 2p3/2 and Fe 2p1/2 were located at ~709.2 and ~722.8 eV, respectively, for clarification of the typical structure of Fe_3_O_4_ as shown in Figure 6d [30−32]. The general spectrum and high-resolution spectra of XPS showed that the MOCC nanocomposite was successfully synthesized from chitosan.

### 3.2. Effect of dose and concentration on drug loading capacity

**Figure 7:**
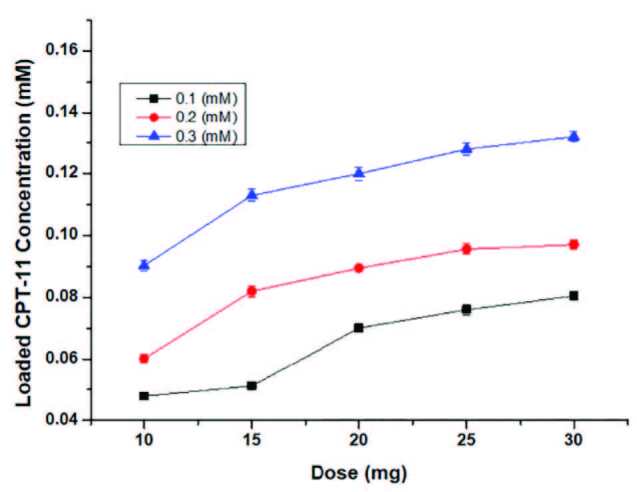
The effect of dose and concentration on CPT-11 loading capacity.

To determine the effect of concentration and dose on drug loading capacity was studied with 3 different concentrations (0.10, 0.20, and 0.30 mM) and 5 different doses (10, 15, 20, 25, and 30 mg). The results were shown in Figure 7. According to the results, the drug loading capacity increased when increased concentration and dose. However, the MOCC nanocomposite highly agglomerated due to magnetic property. To avoid a problem with ingestion of cellular in cytotoxicity experiments, the appropriate dose was determined to be 10 mg in this study. On the other hand, the loaded drug concentration increased when the concentration of CPT-11 was increased (Figure 7). According to the results in Figure 6a, the drug loading capacity of MOCC was found to be quite high. In general, in cytotoxicity studies of CPT-11, the drug concentrations were carried out at the μM level in the literature. Therefore, 0.1 mM initial concentration of CPT-11 was considered suitable for this study [4]. 

### 3.3. Effect of contact time on drug loading efficiency (%)

The drug loading efficiency (%) decreased when the interaction time between the drug and the surface was increased. This situation might be explained by the fact that the loading of CPT-11 on the MOCC occurs through physical interactions such as the hydrogen bonds, van der Waals, and dipole interactions [2]. Therefore, some part of the loaded drug is released from the surface when the interaction time was increased. As a matter of fact, in our previous study, we observed a similar situation in topotecan loading on the nanocomposite [2]. The results were shown in Table.

**Table:** The results of CPT-11 loading efficiency (%) and capacity (mg/g) onto MOCC (amean, bstandard deviation (n = 3)).

Time (min)	CPT-11 loadingefficiency (%)	Loaded CPT-11(mg/g)a	SDb
1	47.87	5.600	0.0018
5	47.00	5.499	0.0016
10	46.20	5.405	0.0014
15	46.00	5.382	0.0015
20	45.80	5.358	0.0014
25	45.60	5.335	0.0017
30	45.10	5.276	0.0015

According to Table, the drug loading capacity (mg/g) decreased when interaction time between MOCC and CPT-11 was increased. After 30 min, the drug loading efficiency (%) decreased by 2.77%. Therefore, the optimum interaction time was limited to 1 min. 

### 3.4. Results of cytotoxicity studies

**Figure 8:**
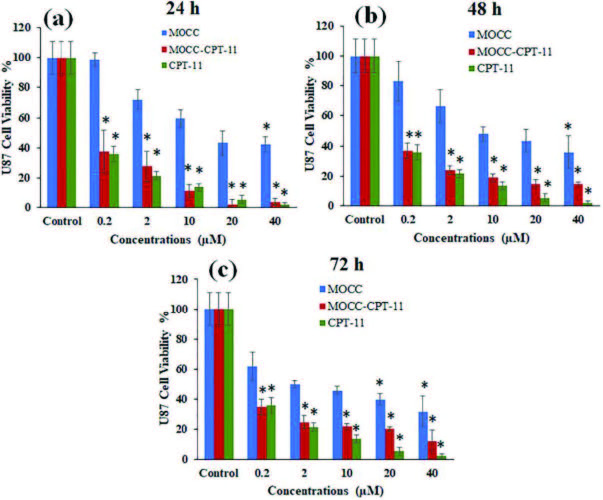
Cell viability of U87 exposed to MOCC, MOCC-CPT-11, and CPT-11 at different concentrations (the significantly different was analyzed with student t-test *p < 0.05).

**Figure 9:**
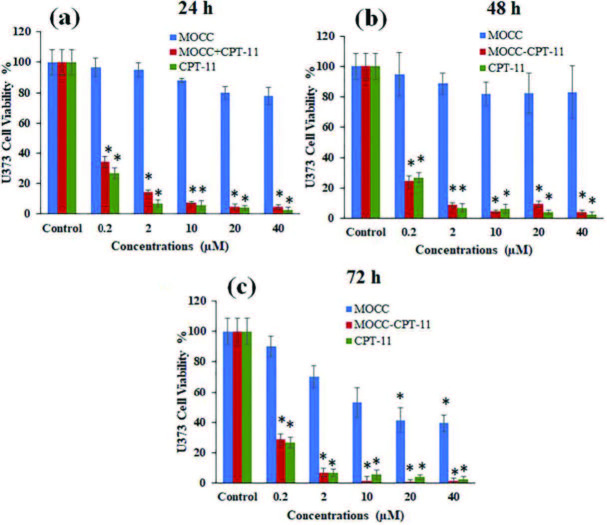
Cell viability of U373 exposed to MOCC, MOCC-CPT-11 and CPT-11 at different concentrations (the significantly different was analyzed with student t-test *p < 0.05).

In Figure 8 and Figure 9, the comparative cytotoxicity assessment of MOCC, MOCC-CPT-11, and free CPT-11 in increasing concentrations (0.2, 2, 10, 20, and 40 μM) and incubation times on the U87 and U373 cell lines were given, respectively. While Figure 8 showed cytotoxicity assessment of MOCC, MOCC-CPT-11, and free CPT-11 on the U87 cells for long incubation times, Figure 9 showed that on the U373 cells. It was observed slight toxicity in cell viability as seen in Figures 8a and 8b when the U87 cell line was exposed to the MOCC at low concentrations, and these values were not found to be statistically significant at low concentrations for all incubation times. However, decreases in cell viability of U87 were found to be statistically significant with increasing MOCC concentrations and incubation times. On the other hand, the cell viability in parallel significantly decreased for all incubation times when the U87 cells exposed to the MOCC-CPT-11 and free CPT-11 in increasing concentrations (see, Figures 8a, 8b, and 8c). Also, MOCC-CPT-11 decreased the cell viability of the U87 cells almost as much as free CPT-11. Furthermore, decreases of cell viability on the U87 cells even at low concentrations of MOCC-CPT-11 were found to be statistically significant in all incubation times.

Besides, the cell viability only slight toxicity showed for 24 h, and 48 h incubation times when the U373 cells were exposed to the MOCC in the range of 0.2 and 40 μM concentration in Figures 9a and 9b. However, for 72 h incubation time, the cell viability decreased from 90% to 39% with increasing concentrations of the MOCC, and it was found statistically significant as seen in Figure 9c. Moreover, the cell viability of U373 noteworthy decreased when the U373 cells were exposed to the MOCC-CPT-11 and free CPT-11 for each concentration and incubation time as seen in Figure 9. Also, the results showed MOCC-CPT-11 to be at least as toxic as free CPT-11 on the cell viability of the U373, and the cell viability values were found statistically significant for each concentration in Figures 9a–9c. These results demonstrate the CPT-11 loaded MOCC for the glioma multiforme, especially for the U373 cells, can be promising in cancer therapy. 

## 4. Conclusion

This study aims to develop an alternative solution to studies on drug load and release systems, which aim to solve problems arising from cancer chemotherapy. Many critical parameters such as drug loading capacity, nanoparticle sizes, and magnetic property of the composite need to be considered when magnetic nanocarrier systems are being designed [3]. For loading the CPT-11, the magnetic featured MOCC was synthesized and characterized using imaging and spectroscopic techniques. The results show that thanks to the carboxyl groups, the MOCC can be used as an excellent drug delivery system with its high drug loading capacity for water-soluble drugs. Moreover, owing to its magnetic feature, it can increase the therapeutic effect of the drug and reduce its toxic effect. In addition, the cytotoxicity results show that the MOCC-CPT-11 significantly decreased the cell viability, especially on the U373 cells, at least as much as free CPT-11. In this study, the cell viability of the U373 decreased to below 1% at some concentration values. Furthermore, the cytotoxicity results of MOCC-CPT-11 on U373 cells have been found to extremely remarkable when compared with the literature. In addition to these, it was found to be suitable for the drug delivery systems owing to the magnetic property and particle size of the MOCC. 

## Funding details

This project is funded by Mardin Artuklu University Research Fund (MAUBAP, project no. MAÜ.BAP.18.SHMYO.001).

## Disclosure statement

No potential conﬂict of interest was reported by the author.
